# Study of anatomical parameters and intraoperative fluoroscopic techniques for transiliac crest anterograde lag screws fixation of the posterior column of the acetabulum

**DOI:** 10.1186/s13018-023-04208-3

**Published:** 2023-09-18

**Authors:** Yangyang Sun, Jian Chen, Fanxiao Liu, Zhixue Chen, Xiaohui Li, Fuxin Lv

**Affiliations:** 1 Department of Orthopaedic Surgery, Qilu Hospital (Qingdao), Cheeloo College of Medicine, Shandong University, 758 Hefei Road, Qingdao, 266035 Shandong China; 2 Department of Orthopaedic Surgery, Shandong Provincial Hospital Affiliated to Shandong First Medical University, Jinan, Shandong China

**Keywords:** Anatomical parameters, Transiliac crest, Acetabulum, Anterograde lag screws, Internal fixation

## Abstract

**Background:**

Currently, there is a lack of research investigating the feasibility of employing anterograde lag screw fixation through the iliac crest for minimally invasive percutaneous treatment of the posterior acetabular column, which encompasses retrograde and anterograde screw fixation in posterior acetabular lag screw fixation. And consequently, the purpose of this study was to examine the anatomical parameters of anterograde lag screw fixation of the posterior column of the pelvis through the iliac crest as well as to investigate the intraoperative fluoroscopy technique, to furnish a scientific rationale supporting the practical utilization of this method within clinical settings.

**Methods:**

In this study, pelvic CT data of 60 healthy adults, including 30 males and 30 females, were accumulated. The mimics 21.0 software was developed to reconstruct the three-dimensional pelvis model, simulate the anterograde lag screw fixation of the posterior column of the acetabulum through use of the iliac crest, and precisely identify the insertion point: Utilizing the widest iliac tubercle as the starting point, the insertion point was moved toward the anterior superior iliac spine by 1.0 cm at a time until it reached 4.0 cm. With a total of five insertion points, all oriented toward the lesser sciatic notch, the initial diameter of the virtual screw measured 5.0 mm, and it was progressively enlarged by 1.0 mm increments until reaching a final diameter of 8.0 mm. Besides, the longest lengths of virtual screws with distinct diameters at divergent entry points were measured and compared. At the same time, the intraoperative fluoroscopy technique for optimal access was analyzed.

**Results:**

The cross-section from the iliac crest to the lesser sciatic notch was irregular, with multiple curved shapes. Furthermore, the diameter of the screw was determined by the anteroposterior radians and width of the iliac crest plate, while the screw length was determined by the curvature of the square body. On the condition that the screw diameter of the D channel (3.0 cm outward from the widest part of the iliac tubercle to the lesser sciatic notch) was 5 mm, 6 mm as well as 7 mm, the longest screw lengths were (145.6 ± 12.8) mm, (143.6 ± 14.4) mm and (139.9 ± 16.6) mm, correspondingly, indicating statistically substantial distinctions from other channels (*P* < 0.0001). Intraoperative fluoroscopy demonstrated that the C-arm machine was tilted (60.7 ± 2.9) ° to the iliac at the entrance position and perpendicular to the D-channel at the exit position.

**Conclusion:**

It is possible to use the new channel to fix the posterior column of the acetabulum with an anterograde lag screw through the iliac crest. In specific, the channel is 3.0 cm outward from the widest part of the iliac tubercle to the lesser sciatic notch. Providing a wide channel, long screw insertion, and high safety, this technique offers a novel approach for minimally invasive treatment of posterior column fractures of the acetabulum.

## Introduction

As a result of the advancements made in fluoroscopy and computer navigation technology, scholars have utilized lag screw fixation as a treatment modality for fractures affecting the posterior acetabular column [1, 2]. This approach provides numerous advantages, namely, lessened soft tissue dissection, minimal blood loss, and reliable fixation [3, 4]. Moreover, the treatment modalities of lag screw fixation of the posterior acetabular column include retrograde and anterograde screw fixation.

Based on the previous research, the entry point of the percutaneous retrograde lag screw is the sciatic tuberosity [5]. The majority of the literature reported [6, 7] that on the condition that the patient is in the supine position and retrograde lag screws are placed, the patient needs to fully flex the hip and knee to effectively reach the sciatic tuberosity and specify the nail entry point while avoiding injury to the sciatic nerve. A prone or lateral position has also been reported [5].

Currently, the entry point for anterograde lag screws is typically located at the iliac fossa. Mu et al. [8] studied the axis of the posterior column on the inner table of the iliac wing and determined that the most advantageous entry point for anterograde lag screws for fixation of the posterior column of the acetabulum is located at (16.8 ± 2.1) mm from the arch rim and (23.5 ± 3.4) mm from the anteriormost portion of the sacroiliac joint. Besides, Shahulhameed et al. [9] measured the entry point sites of the anterior and posterior columns of the acetabulum in 11 adult pelvic cases and asserted that the mean distance of the posterior column anterograde lag screw entry point was 3.11 cm from the arch rim and 3.96 cm from the sacroiliac joint. For complex fractures, including T-shaped fractures, bicolumnar fractures, as well as anterior column plus posterior hemi-transverse fractures, fixation of the fracture with an anterior column plate plus posterior column screw fixation upon repositioning the posterior column through a single anterior approach is an ideal fixation modality [10].

The iliac crest, which exhibits an upward convexity in an "S" shape, represents the upper border of the iliac bone. Positioned approximately 5–7 cm away from the anterior superior iliac spine, the iliac tuberosity protrudes outward and can be readily palpated on the body surface. The bony segment spanning between the anterior superior iliac spine and the iliac tuberosity is characterized by its considerable width and abundant bone content.

Can percutaneous minimally invasive treatment of the posterior column of the acetabulum be accomplished by utilizing anterograde lag screws inserted through the iliac crest to fixate the posterior column of the acetabulum, and if so, where is that optimal access? Consequently, this study was designed and divided into three parts. The study also examined intraoperative fluoroscopic techniques for achieving optimal access, and the results of these investigations are presented below.

## Materials and methods

### Data acquisition

The study protocol was approved by the Ethics Committee of Qilu Hospital (Qingdao), Cheeloo College of Medicine, Shandong University, and all participants in the study signed an informed consent form in writing. Sixty normal adults, 30 of each sex, with an average age of 45.2 years (19–65 years) for males and 44.7 years (20–60 years) for females, who underwent electron computed tomography (including whole pelvis CT) in the 64-row CT room between September 2022 and April 2023, were randomly sampled. Inclusion criteria: (1) normal adult pelvis, 18 years ≤ age ≤ 70 years; (2) complete imaging data. Exclusion criteria: 1. age < 18 years or age > 70 years; 2. bone and ligament injury of the pelvic ring, acetabular fracture; (3) primary or secondary pelvic malignancy; (4) bone metabolic disease; (5) severe bone and joint degeneration; (6) previous pelvic and acetabular fracture; (7) previous pelvic and acetabular fracture treated surgically, etc.

## Three-dimensional reconstruction of the pelvis

The underwent continuous spiral CT scanning on the abdomen, with voltage of 120kv, slice thickness of 0.625 mm and matrix of 512 × 512. The CT images were preserved in.dicom format and imported into the Mimics 21.0 software (Materialize, Belgium). Subsequently, the standard adult bone thresholds (CT values between 226  and 1542 Hu) were used. Three-dimensional model of the pelvis was reconstructed successfully.

## Measured data

**Fig. 1:**
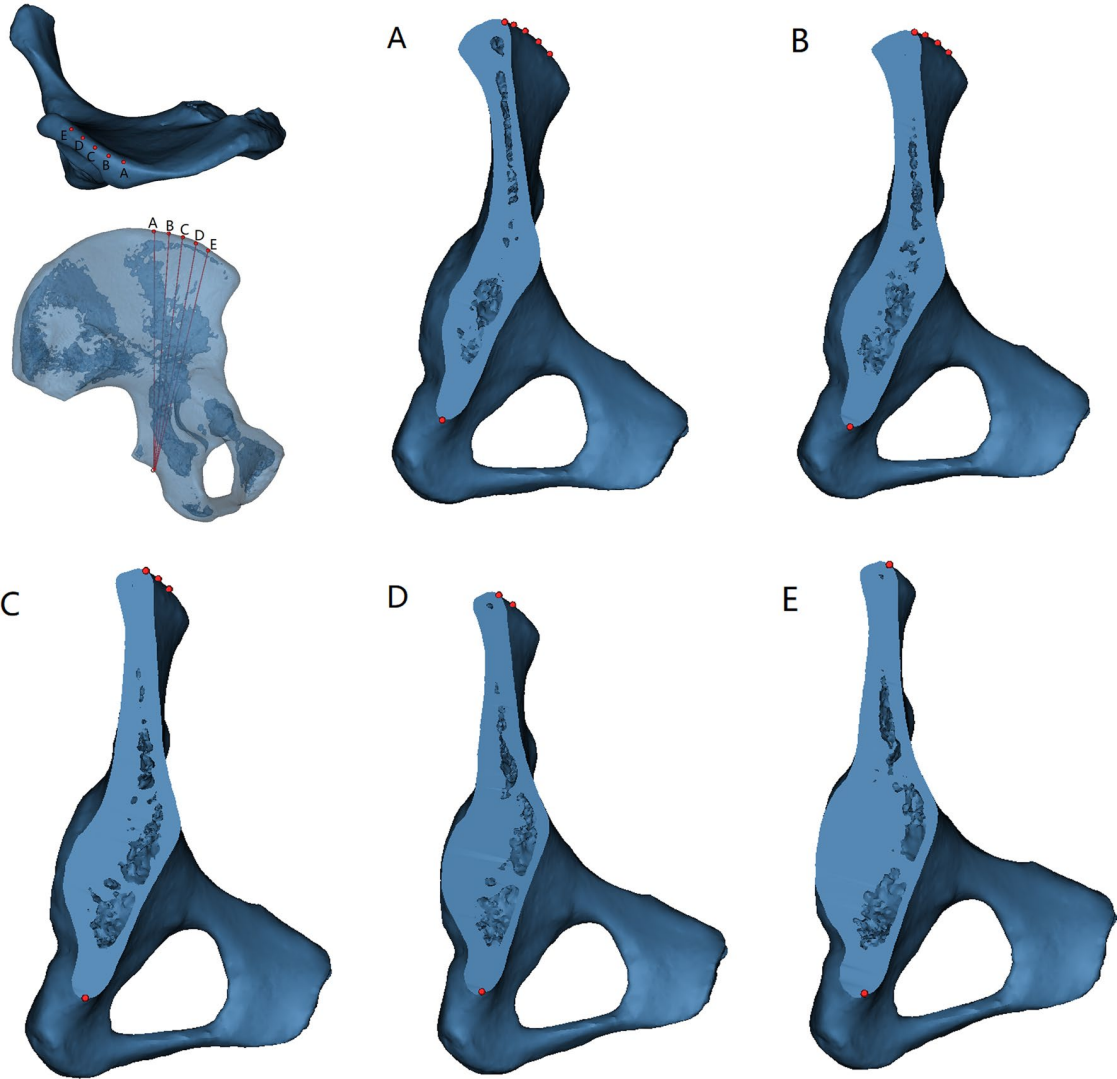
The widest point of the iliac tuberosity is adopted as the starting point, and the nail is increased by 1.0 cm each time in the direction of the anterior superior iliac spine until it reaches 4.0 cm in height, which is divided into 5 nailing points: point **A** (the widest point of the iliac tuberosity), point **B** (the widest point of the iliac tuberosity 1 cm outward), point **C** (the widest point of the iliac tuberosity 2 cm outward), point **D** (the widest point of the iliac tuberosity 3 cm outward), point **E** (the widest point of the iliac tuberosity 4 cm outward). **A**, **B**, **C**, **D**, and **E** represent the cross-sections from point A, point B, point C, point D, and point E to the lesser sciatic notch, respectively

The 3D model of the pelvis was reconstructed to simulate the fixation of the posterior column of the acetabulum through the iliac crest with an anterograde lag screw, and the nailing points were positioned: Starting from the widest point of the iliac tuberosity, the nailing points were increased by 1.0 cm in the direction of the anterior superior iliac spine until they reached 4.0 cm, which was divided into 5 nailing points (point A was the widest point of the iliac tuberosity; point B was the widest point of the iliac tuberosity 1 cm outward; point C was the widest point of the iliac tuberosity 2 cm outward; point D represented 3 cm outward from the widest part of the iliac tuberosity; point E symbolized 4 cm outward from the widest part of the iliac tuberosity), oriented toward the lesser sciatic notch (Fig. 1).

**Fig. 2:**
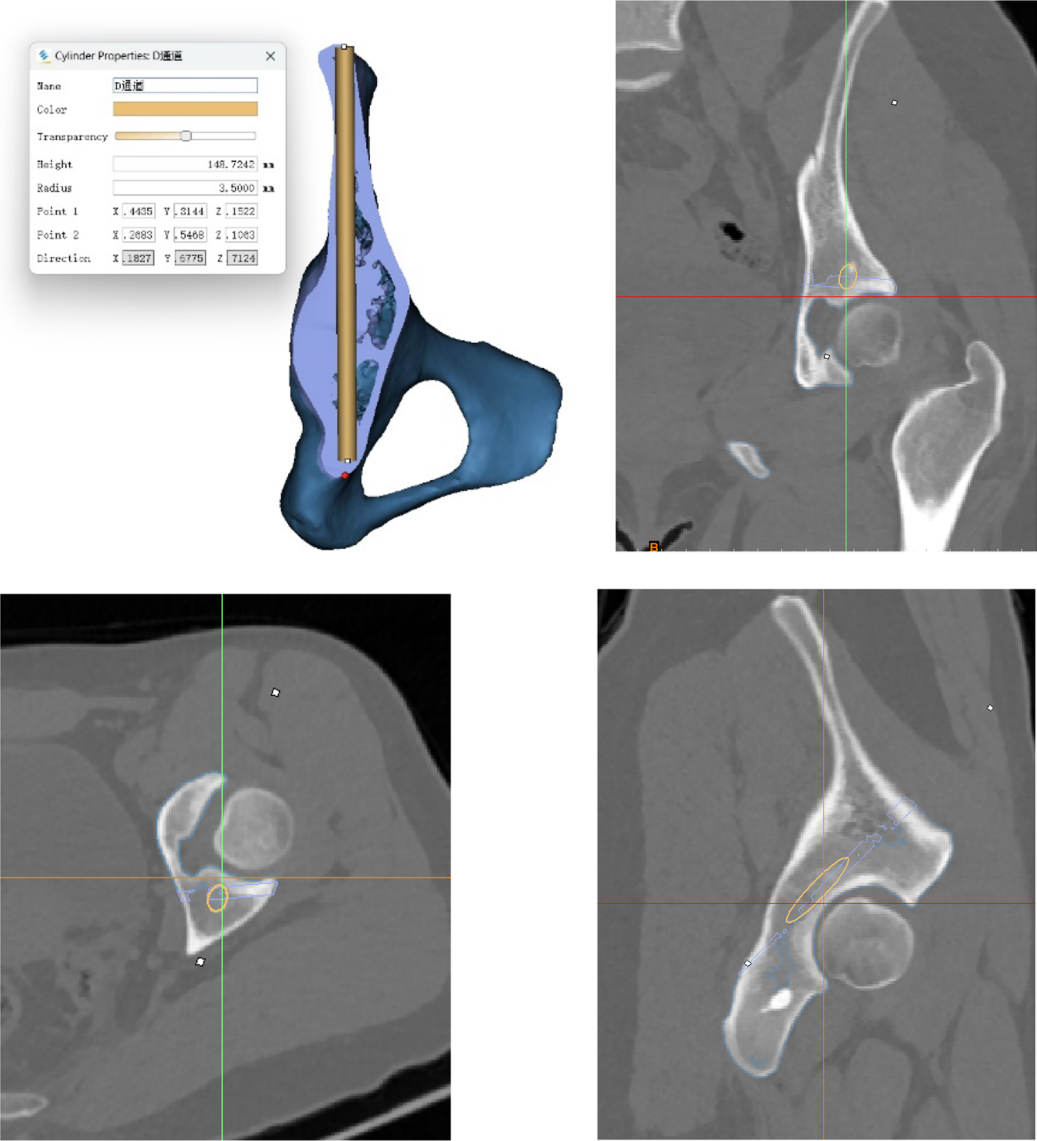
A three-dimensional pelvis model was created, which simulated the fixation of the posterior column of the acetabulum through the iliac crest using anterograde lag screws. The screws were directed toward the lesser sciatic notch and positioned in the coronal, horizontal, and sagittal planes. The virtual screws were securely placed within the bone, without penetrating the bone cortex or entering the acetabulum

Each channel makes a cross-section, which is inconsistent in form and has multiple curved arcs (Fig. 1). Besides, four elements determine the diameter and length of the screw; the screw diameter is determined by the anterior–posterior curvature and width of the iliac plate. Moreover, the curvature of the square-shaped body determines the length of the screw (Fig. 1). Except for that, the present investigators chose virtual tension screw placement with the smallest diameter of 5.0 mm and measured the longest length that may be positioned at distinct entry points, and increased 1.0 mm each time thereafter until a screw with a diameter of 8.0 mm (Figs. 1 and 2).

The posterior acetabular column lag screws were screened using three-dimensional reconstruction and three distinct flat films, including the sagittal, coronal, and horizontal planes of the pelvis (Fig. 2). During the simulated procedure, the lag screw was deemed to have failed in the following three circumstances and rejected: (1) the screw penetrated the lateral bone cortex within the square; (2) the screw entered the joint cavity and ruined the surface of the joint; and (3) the screw penetrated the bone plate on either side. A well-positioned screw is positioned in the bone from the point of entry to the point of exit.

## Data measurement and processing

The measurement process was performed in pairs and the average was calculated to eliminate subjectivity from the data. Besides, the prerequisites were that the measurers were proficient in the purpose, principles, and imaging fundamentals of the experiment and operation of the Mimics 21.0 software. Moreover, the measurement data were recorded utterly and the results were statistically analyzed utilizing SPSS 20.0 statistical software (IBM SPSS Statistics 20.0, IBM Co, us). The mean and standard deviation ($\overline{x }\pm s$) of each parameter was calculated, and the F-test was performed on data within the same group and between various groups, with *a* = 0.05 as the test level.

## Results

### Maximum length of virtual screws of different diameters at different nail entry points

The results of the obtained channels showed that the smaller the diameter of the virtual screws, the longer the lengths that could be placed (Tables 1 and 2). At screw diameters of 5.0 mm, 6.0 mm, and 7.0 mm, the lengths of the placeable screws in channel D were (145.6 ± 12.8) mm, (143.6 ± 14.4) mm, and (139.9 ± 16.7) mm, respectively, with statistically significant differences compared with the other channels (*P* < 0.0001). When the diameter of the screw was 8 mm, the length of the E channel was the longest, and the difference was statistically significant compared with other channels (*P* < 0.0001), however, the E channel was closer to the acetabulum, and some of the virtual screws, especially those with larger diameters, easily penetrated into the acetabulum. If the 8.00 mm screw in the E channel was excluded, the difference between the longest screw in the D channel at 8.00 mm and the other channels was also statistically significant (*F* = 3.772, *P* = 0.0124). One female pelvic model (1/60) had screws that were too short to achieve fixation successfully.

**Table 1:** Maximum length of virtual screws with distinct diameters for divergent nail entry points(mm,($\overline{{\varvec{x}}}\pm {\varvec{s} }$))

	5	6	7	8
Channel A	123.9 $\pm$ 18.2	122.8 $\pm$ 17.3	118.9 $\pm$ 15.3	117.5 $\pm$ 13.2
Channel B	132.0 ± 19.3	128.7 $\pm$ 18.9	127.9 $\pm$ 20.0	124.8 $\pm$ 17.9
Channel C	141.2 $\pm$ 16.2	138.9 $\pm$ 17.8	134.5 $\pm$ 18.9	129.6 $\pm$ 20.8
Channel D	145.6 $\pm$ 12.8	143.6 $\pm$ 14.4	139.9 $\pm$ 16.7	134.3 $\pm$ 19.8
Channel E	142.5 $\pm$ 13.1	140.9 $\pm$ 14.5	137.1 $\pm$ 17.4	138.4 $\pm$ 18.4
*F*	12.9	10.7	7.2	4.3
*P*	< 0.0001	< 0.0001	< 0.0001	0.0025

**Table 2:** Maximum lengths of virtual screws of distinct diameters for channel D(mm, ($\overline{{\varvec{x}}}\pm {\varvec{s} }$))

	5	6	7	8
Channel D	145.6 $\pm$ 12.8	143.6 $\pm$ 14.4	139.9 $\pm$ 16.7	134.3 $\pm$ 19.8
*F*	4.1			
*P*	0.008			

## Intraoperative fluoroscopic technique

**Fig. 3:**
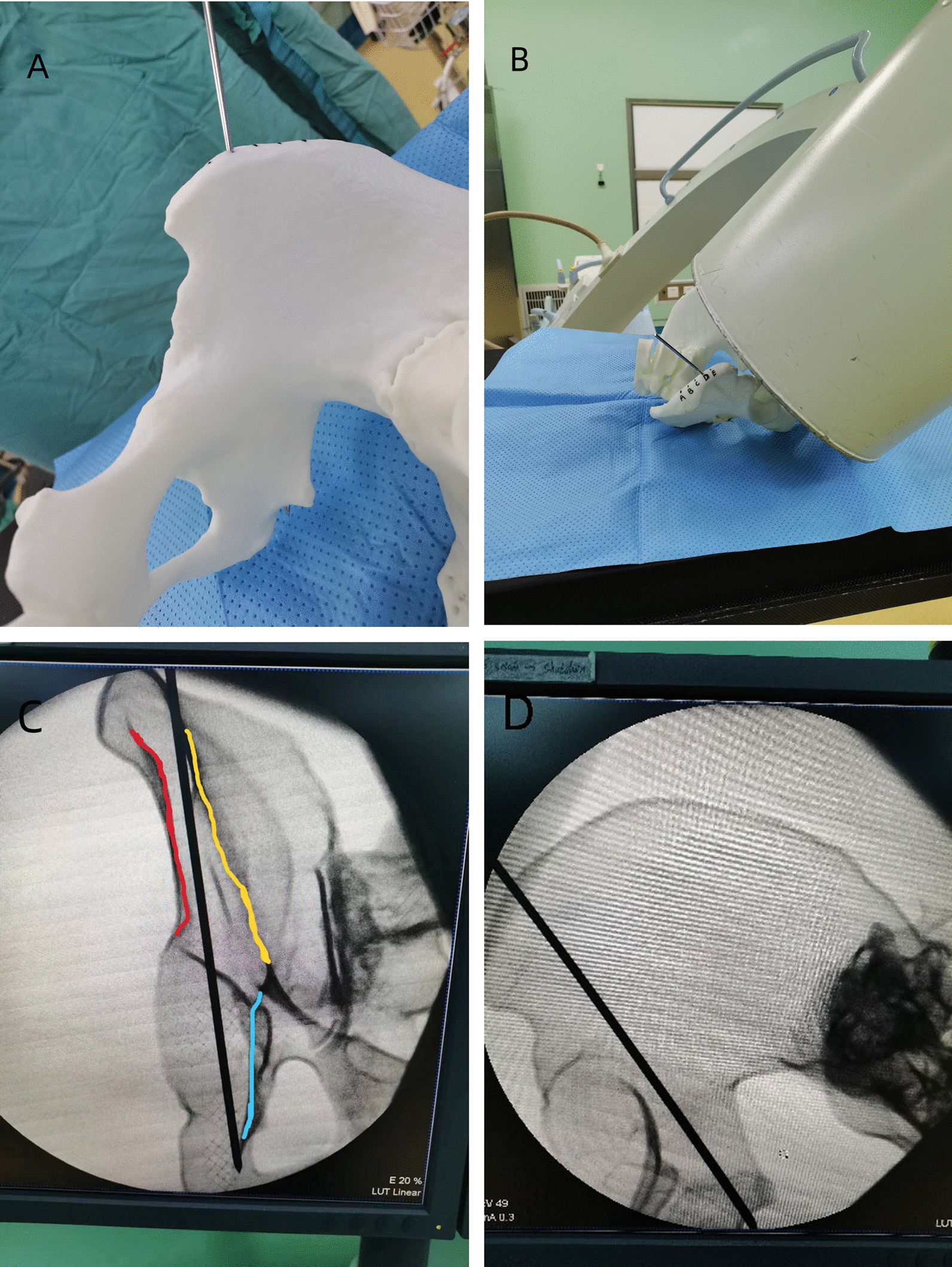
**A** indicates the 3D printed pelvic specimen using a 3.00 mm Kirschner wire placed in the D channel; **B** demonstrates the intraoperative fluoroscopic C-arm machine with the pelvic specimen; **C** illustrates the lateral position and the full length of the Kirschner wire in the exit position and perpendicular to the D channel. The red line represents the posterior edge of the iliac plate, the yellow line represents the anterior edge of the iliac plate, and the blue line represents the inferior edge of the greater sciatic notch. There is no screw penetration of the bone cortex anterior to the inferior edge of the greater sciatic notch, where the screw is placed. **D** reveals the full length of the frontal Kirschner wire in the entrance position with iliac oblique 60° fluoroscopy

Thirty cases of normal adult pelvic CT data were selected (15 of each sex, with an average age of 44.7 years for males and 45.3 years for females), 3D printed pelvic specimens were simulated in the horizontal position, as well as the investigator selected a 3.00 mm Kirschner wire, using point D and oriented toward the lesser sciatic notch as the entry point, the incision is made and placed the Kirschner wire under the guidance of an X-ray C-arm machine (Fig. 3). Besides, the study concluded that the fluoroscopic position was the entrance position of the iliac bone oblique (60.7 ± 2.9)° to allow fluoroscopic examination of the entire length of the Kirschner's wire; the exit position perpendicular to the D channel (where the C-arm machine is located) supposed to allow fluoroscopy of the lateral Kirschner's wire position and length.

## Discussion

The cross-section from the iliac crest to the lesser sciatic notch indicates an irregular shape, and the iliac plate contains two curves: the anterior cortical bone is concave, the posterior cortical bone is convex, the arch is more prominent at the arch line, and the quadrilateral body is oblique. The diameter and length of the screw are determined by four factors: the anterior–posterior curvature of the iliac plate and the width of the iliac plate determine the screw's diameter, while the slope of the quadrilateral body determines the screw's length. This channel is significantly more complex than the other channels. Besides, the anatomy of the transiliac fossa in a cascade or retrograde through the iliac tuberosity reveals the existence of a solid bony channel in the posterior column of the acetabulum that is approximately “trigonous” in shape [11].

Hence, direct measurement of the longest length of the section is not a helpful reference for the screw's actual clinical placement. In this study, the analysis of the five access cross-sections illustrated that the more outward the access point, the less anterior and posterior curvature of the iliac plate, the less slope of the square body, and the length of the virtual screw that can be placed is proportional to the width of the iliac plate. Present results are comparable with those by other authors. The Morandi [12] explored the narrowing ranges of safe insertion with increasing screw lengths in the supraacetabular area and the relationship between anatomical size of the pelvis and screw size. The author concluded that the 60 mm screw depth provided the best option to limit breaching of the pelvic wall having the largest range in both transverse and sagittal directions. Solitro [13] developed a method and mathematical lemmas that were fundamental to the development of computer algorithms for pedicle screw placement.

The nail entry point in this study varies from the findings of other scholars [14–16] in that the focus of this study was to investigate percutaneous transiliac crest anterograde lag screw fixation of the posterior column of the acetabulum. Besides, the nail entry point in this study was located between the iliac tuberosity and the anterior superior iliac spine, which possessed the benefits of simpler positioning and less trauma, and was oriented toward the lesser sciatic notch. In clinical application, the iliac tuberosity is employed as a body marker, and in obese patients, where it is occasionally challenging to palpate the iliac tuberosity, the anterior superior iliac spine may be selected as a body marker. The result of this study demonstrates that the length of the virtual screws of distinct diameters that can be placed in channel D is extremely long, and the distinction is statistically significant in contrast to other channels. Within the scope of this study, certain instances were encountered wherein the placement of virtual screws proved challenging. Additionally, in cases where the smallest diameter screws were employed and their length was relatively short, the desired outcome of achieving optimal bone fixation was not attained.

Mouhsine et al. [17] adopted 7.3 mm lag screws to fix the anterior and posterior columns of the acetabulum and achieved satisfactory clinical outcomes in all patients during follow-up, concluding that 7.3 mm lag screws could provide sufficient fixation force. Starr et al. [18] studied percutaneous fixation of the anterior and posterior columns of the acetabulum and recommended that 7.3 mm or 8.0 mm lag screws could be employed for the posterior column of the acetabulum. Jung et al. [19] studied 178 virtual 3D models of the hemipelvis and concluded that the safety zone for posterior acetabular column placement screws was triangular with a mean maximum cylindrical diameter of 7.4 mm. As previously mentioned, due to the irregular shape of the access, the authors began with a minimum diameter of 5 mm and gradually increased it by 1 mm until it reached 8 mm. Moreover, the access was safe and effective for screws of distinct diameters in three views, sagittal, coronal, and cross-sectional, completely within the bone without penetrating the bone cortex and penetrating the acetabulum, and entering the joint. When the diameter is reduced, the maximum length of the screw that can be inserted is also increased. Moreover, when the minimum diameter of the virtual screw was 5.0 mm, the maximum length of the D channel was (145.6 ± 12.8) mm; when the maximum diameter of the virtual screw was 8.0 mm, the maximum length of the D channel was (134.3 ± 19.8) mm. Due to pronounced anterior and posterior curvatures of the iliac plate and/or limited width of the iliac plate, one female pelvic model (1.6%) used in this research had screws that were too short to achieve fixation, which caused the fixation to be unsuccessful. Preoperative planning is extremely crucial to select the appropriate diameter and length of screws on the basis of the shape of the pelvis and the location of the fracture line.

The results of this study demonstrated that the access point of the widest iliac tuberosity 3 cm outward, with the direction pointing to the lesser sciatic notch, was the most convenient access. To simulate intraoperative fluoroscopy, during screw placement, fluoroscopic positions, including pelvic orthostatic, iliac oblique 45° and closed-hole oblique 45°, are commonly employed for the purpose of determining the direction of screw entry and whether the screw enters the joint or penetrates the bone cortex, contributing to internal fixation failure. Nevertheless, the D-channel structure of the posterior column of the fixed acetabulum is irregular, and the above fluoroscopic positions cannot completely reflect whether the screw penetrates the posterior wall of the acetabulum and the quadrilateral area, which is adjacent to the sciatic nerve, supra pelvic vessels and internal pelvic organs, and any injury will lead to serious complications. So, thirty of 3D-printed pelvic specimen models were utilized in this study to insert screws into the D-channel safely and effectively during simulated surgery. It was observed that when the iliac oblique position was set at (60.7 ± 2.9) ° for the entry point and the exit point was positioned perpendicular to the D-channel, the fluoroscopy position of the C-arm machine enabled clear visualization of the position and length of the Kirschner wire.

Additionally, we determined that the indications for minimally invasive surgery using transiliac crest paralleling tension screws are as follows: (1) transverse acetabular fractures or T-shaped fractures without displacement or displacement less than 2.00 mm, etc.; (2) posterior acetabular column fractures can be securely placed with paralleling tension screws upon closed repositioning so that the fracture ends can be repositioned with displacement less than 2.00 mm; (3) despite the evident displacement in acetabular fractures, the fracture of the anterior column can be reduced through a straightforward anterior approach, while the fracture of the posterior column can also be fully or partially reduced, allowing for secure fixation using parallel lag screws.

There are some limitations to our research. One limitation is that this study used intact pelvises for reconstruction, and the research approach was similar to that of other scholars. Young et al. [20] sectioned the pelvis by reconstructing three-dimensional model of the pelvis to determine the greatest degree of sagittal pin spanning angulation between two iliac crest pins and how intraosseous depth affects these angulations. Morandi et al. [12] also designed the study that A computer algorithm created cross‐sections over three‐dimensional pelvic reconstructions to quantify the narrowing of safe supra‐acetabular pin corridors in the transverse and sagittal planes with increments of intraosseous screw depths. All the measures we performed were based on different entry points. And we did not evaluate the sensitivity of our results to the variation of entry point. These virtual screws do not exist, and due to their long length, require specially customized. Another limitation is represented by the adoption of radiographic CT scans in place of cadaveric specimens. it was more feasible to analyze these planes of virtual screws using computer simulations that allowed the sagittal, coronal and horizontal planes to be displayed at once. However, Anatomical specimens better reflect the real situation. Haidukewych et al. [21] examined the placement of half-pins for supra-acetabular external fixation in ten fresh frozen cadaveric pelvises. Apivatthakakul et al. [22] selected fifteen fresh-frozen non-preserved whole body anatomical specimens with an intact pelvis and no previous surgery in the abdominal area to identify structures at risk after application of anterior subcutaneous pelvic internal fixator. So cadaveric validations of our results can be tested for further investigation. The next step is to examine the mechanical characteristics of the channel through biomechanical and finite element analysis and to conduct research on the biomechanical differences between the channel and the conventional nail placement channel in order to supply a more solid scientific basis for clinical application. For the purpose of solving the issue of how to apply the data obtained from the experiments to the clinic, and to study the nail placement guide, which will increase the reliability of the procedure while simultaneously expanding its use in medical settings.

## Conclusion

The new channel is feasible for the fixation of the posterior column of the acetabulum via the iliac crest with paralleling tension screws. In specific, the access (3.0 cm outward from the widest point of the iliac tuberosity to the lesser sciatic notch), which is wide, long enough to accommodate the placement of screws, and secure, provides a new treatment modality for minimally invasive treatment of posterior acetabular column fractures.

## Data Availability

The datasets used and analyzed during the present study are available from the author on reasonable request.

## References

[1] Chen H, Wang G, Li R (2016). A novel navigation template for fixation of acetabular posterior column fractures with antegrade lag screws: design and application. Int Orthop.

[2] Chui KH, Chan CCD, Ip KC (2018). Three-dimensional navigation-guided percutaneous screw fixation for nondisplaced and displaced pelvi-acetabular fractures in a major trauma centre. Int Orthop.

[3] Cavalié G, Boudissa M, Kerschbaumer G (2022). Clinical and radiological outcomes of antegrade posterior column screw fixation of the acetabulum. Orthop Traumatol Surg Res.

[4] Ye J, Xie L, Liu Z (2021). Anterograde lag screw placement in the posterior column of the acetabulum: a case report and literature review. Trauma Case Rep.

[5] Levin S, Krumins R, Shaath MK (2022). Clinical outcomes in prone positioning for percutaneous fixation of posterior column acetabular fractures. Eur J Trauma Emerg Surg.

[6] Caviglia H, Mejail A, Landro ME (2018). Percutaneous fixation of acetabular fractures. EFORT Open Rev.

[7] Bozzio AE, Johnson CR, Mauffrey C (2016). Short-term results of percutaneous treatment of acetabular fractures: functional outcomes, radiographic assessment, and complications. Int Orthop.

[8] Mu WD, Wang XQ, Jia TH (2009). Quantitative anatomic basis of antegrade lag screw placement in posterior column of acetabulum. Arch Orthop Trauma Surg.

[9] Shahulhameed A, Roberts CS, Pomeroy CL (2010). Mapping the columns of the acetabulum–implications for percutaneous fixation. Injury.

[10] Le Quang H, Schmoelz W, Lindtner RA (2021). Single column plate plus other column lag screw fixation vs. both column plate fixation for an anterior column with posterior hemitransverse acetabular fractures - a biomechanical analysis using different loading protocols. Injury.

[11] Feng X, Zhang S, Luo Q (2016). Definition of a safe zone for antegrade lag screw fixation of fracture of posterior column of the acetabulum by 3D technology. Injury.

[12] Morandi MM, Daily D, Kee C (2019). Safe supra-acetabular pin insertion in relation to intraosseous depth. J Orthop Res.

[13] Solitro GF, Amirouche F (2016). Innovative approach in the development of computer assisted algorithm for spine pedicle screw placement. Med Eng Phys.

[14] Azzam K, Siebler J, Bergmann K (2014). Percutaneous retrograde posterior column acetabular fixation: is the sciatic nerve safe?. A cadaveric study J Orthop Trauma.

[15] Yu K, Zhou R, Gao S (2022). The placement of percutaneous retrograde acetabular posterior column screw based on imaging anatomical study of acetabular posterior column corridor. J Orthop Surg Res.

[16] Xu Y, Lin C, Zhang L (2016). Anterograde fixation module for posterior acetabular column fracture: computer-assisted determination of optimal entry point, angle, and length for screw insertion. Med Sci Monit.

[17] Mouhsine E, Garofalo R, Borens O (2005). Percutaneous retrograde screwing for stabilisation of acetabular fractures [J]. Injury.

[18] Starr AJ, Reinert CM, Jones AL (1998). Percutaneous fixation of the columns of the acetabulum: a new technique. J Orthop Trauma.

[19] Jung GH, Lee Y, Kim JW (2017). Computational analysis of the safe zone for the antegrade lag screw in posterior column fixation with the anterior approach in acetabular fracture: a cadaveric study. Injury.

[20] Young B, Daily D, Kee C (2020). Achievable pin spanning angulation in anterosuperior pelvic external fixation. Eur J Orthop Surg Traumatol.

[21] Haidukewych GJ, Kumar S, Prpa B (2003). Placement of half-pins for supra-acetabular external fixation: an anatomic study. Clin Orthop Relat Res.

[22] Apivatthakakul T, Rujiwattanapong N (2016). Anterior subcutaneous pelvic internal fixator (INFIX), Is it safe?. A Cadaveric Study Inj.

